# Hypocalcemia Induced Bronchospasm

**DOI:** 10.7759/cureus.26339

**Published:** 2022-06-26

**Authors:** Aneeta Kumari, Kunal Nangrani, Tsering Dolkar, Amita Arora, Marie Schmidt

**Affiliations:** 1 Pulmonary Medicine and Critical Care Medicine, One Brooklyn Health System, Interfaith Medical Center, Brooklyn, USA; 2 Pulmonary Medicine and Critical Care Medicine, The Brooklyn Hospital Center, Brooklyn, USA; 3 Internal Medicine, One Brooklyn Health System, Interfaith Medical Center, Brooklyn, USA; 4 Pulmonary and Critical Care Medicine, One Brooklyn Health System, Interfaith Medical Center, Brooklyn, USA

**Keywords:** management of hypocalcemia induced bronchospasm, persistent bronchospasm, hypocalcemia induced bronchospasm, hypocalcemia, bronchospasm

## Abstract

Bronchospasm is acute narrowing of the airways of lungs, which gives rise to wheezing and shortness of breath. Commonly seen in obstructive lung disease, but a rare finding in patients with hypocalcemia. This is a case that outlines a rare presentation of hypocalcemia induced bronchospasm in a patient with no known history of asthma or chronic obstructive pulmonary disease (COPD). In this report, we present a case of a 57 years old male with no history of asthma or COPD who presented with intractable bronchospasm. Initial work-up for common entities was negative. Patient was found to be profoundly hypocalcemic and treatment provided resolution of symptoms. Early recognition of hypocalcemia induced bronchospasm is important in clinical practice to optimize management and provide improvement in symptoms.

## Introduction

Bronchospasm is an acute narrowing of the airways of the lungs, which gives rise to wheezing and shortness of breath [1,2]. Patients with known asthma can have reversible bronchospasm, which is precipitated by an inciting factor [2]. On the other hand, patients with chronic obstructive pulmonary disease (COPD) have chronic narrowing of the bronchus and may present with wheezing or shortness of breath during an acute exacerbation [2]. This case is about a rare presentation of hypocalcemia-induced bronchospasm in a patient with no known history of asthma or COPD.

## Case presentation

**Figure 1:**
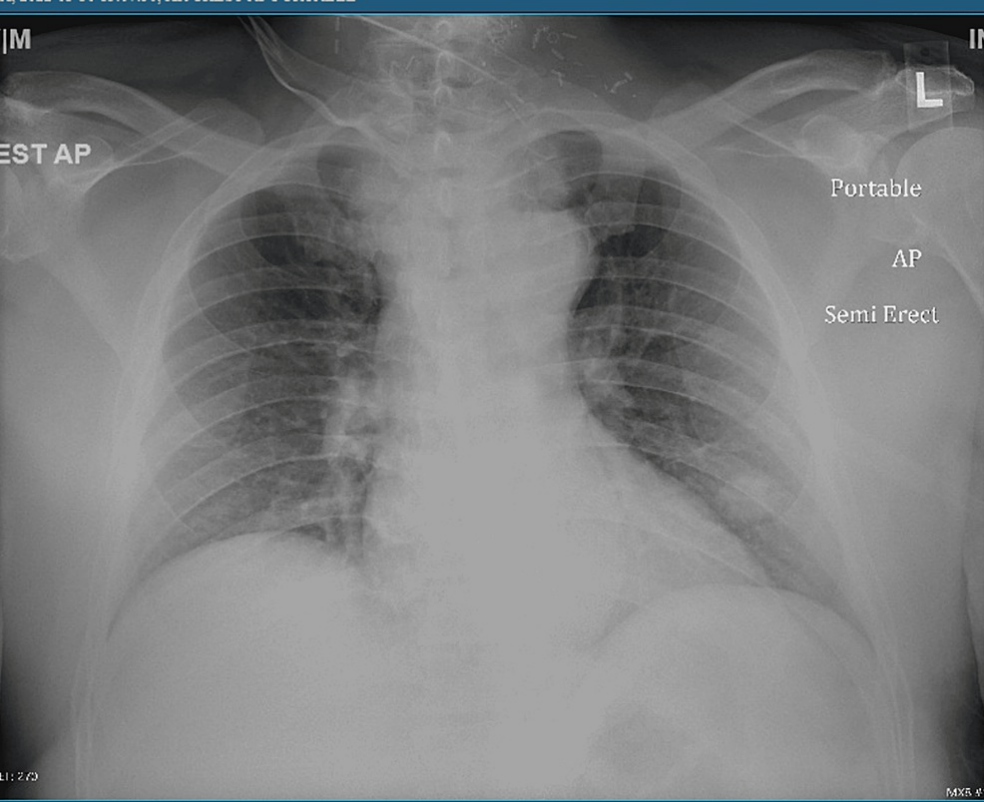
CXR on admission showed lung fields are symmetrically aerated. Tracheostomy tube position somewhat eccentrically to the right. Pneumomediastinum was a new finding after the tracheostomy tube was changed by the surgery team, which was resolved with conservative management. A lung nodule is noted at the left lung base.

A 57 years old male with a past medical history of laryngeal cancer status post neck surgery, tracheostomy, and radiation therapy (in 2019) presented to the Emergency Department (ED) with complaints of progressive shortness of breath for the last 2 to 3 days. On arrival, the patient was tachycardic, tachypneic, and noted to have diffuse wheezing. Initial arterial blood gas (ABG) showed a pH of 7.38 (range 7.31-7.41) with high partial pressure of carbon dioxide (pCO2) of 56 (range 41-51) and normal oxygen saturation. Chest X-ray (Figure 1) done was negative for any consolidation or infiltrates. 

A CT angiography of the chest was performed due to elevated D-dimer, which did not reveal any pulmonary embolism, consolidation, or infiltrates. The patient was started on IV steroids and bronchodilators. However, the patient became more tachypneic and had worsening wheezing. A surgical team was consulted, and the tracheostomy was changed. Subsequently, the patient was placed on mechanical ventilation via trach.

**Figure 2:**
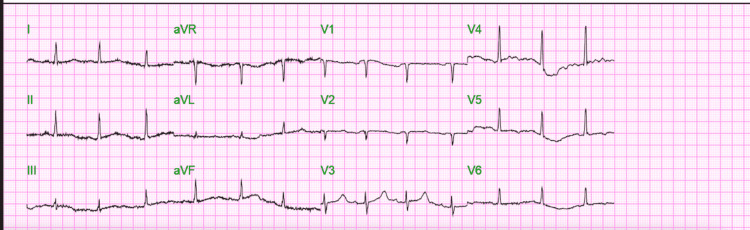
EKG on admission: Normal sinus rhythm with heart rate of 80 with QTc of 507.

Initial labs were normal except for total calcium which was low 6.7mg/dl (range 8.8 to 10.0mg/dl) (Table 1) and lowest ionized calcium was 0.9 mg/dL (range 4.8 to 5.6 mg/dL). Serum albumin and other electrolytes, namely magnesium, phosphorus, and potassium, were normal. PTH was low at <3.4 pg/mL, and vitamin D level was low at 12.8 ng/mL. EKG showed normal sinus rhythm with a heart rate of 80 and a prolonged QTc interval of 507 (Figure 2).

An endocrine team evaluation was done, and the patient was started on IV calcium gluconate and oral calcium. Gradually patient symptoms improved, and he was weaned off the ventilator. IV steroids and bronchodilators were discontinued. Calcium levels improved to 7.9-8 mg/dl with ionized calcium to 5 mg/dL. He was discharged on room air successfully and was started on Teriparatide upon discharge and advised to follow with endocrine as an outpatient. The laboratory investigations on admission are shown in Table 1.

**Table 1:** Laboratory investigations on admission WBC: White blood cells, BUN: Blood urea nitrogen, BNP: Brain natriuretic peptide

Table 1:		
Investigation	Value	Reference range
Hemoglobin	12.3	11.0 - 15.0 g/dL
Hematocrit	36.4	
WBC	5.9	3.8 - 5.3 10x6/uL
Platelets	364	130 - 400 10x3/uL
Glucose	76	80 - 115 mg/dL
BUN	9	9.8 - 20.1 mg/dL
Creatinine	1.05	0.57 - 1.11 mg/dL
Sodium	137	136 - 145 mmol/L
Potassium	4	3.5 - 5.1 mmol/L
Chloride	98	98 - 107 mmol/L
Bicarbonate	23	23 - 31 mmol/L
Calcium	6.7	8.8 - 10.0 mg/dL
Albumin	4.3	3.2 - 4.6 g/dL
Corrected Calcium	6.5	
Magnesium	2.1	1.6 - 2.6 mg/dL
BNP	24	10.0 - 100.0 pg/mL
COVID PCR	Negative	
High sensitivity Troponin I	<3.5	0.0 - 17.0 ng/L

## Discussion

Calcium is important in regulating the membrane permeability of cardiac action potential. Hence any disruption in calcium can lead to cardiac arrhythmias [3]. Four parathyroid glands located in the thyroid tissue regulate the calcium metabolism in the body [4]. The parathyroid glands secrete PTH (parathyroid hormone), which leads to increased serum calcium in the body by increasing calcium release from the bone and decreasing calcium excretion from the kidneys by increasing reabsorption [5]. Parathyroid hormone (PTH) also acts on kidneys to stimulate the formation of vitamin D. The most common cause of hypoparathyroidism is surgical [6]. Excision or damage to parathyroid glands during thyroidectomy or neck surgery for head and neck cancer is well known. This leads to dysregulation of calcium in the body, particularly low calcium levels leading to signs and symptoms of hypocalcemia. Surgeries for thyroidectomy are combined with reimplantation of parathyroid glands subcutaneously in the forearms, which may take weeks to months to become functional [7]. Calvallaro et al. show that 90% have graft functionality after one year of parathyroid gland reimplantation [7]. The typical symptoms of hypocalcemia are paresthesia in the peri-oral and extremities, along with fatigue, anxiety, and tetany. In extreme cases, bronchospasm and laryngospasm with wheeze and stridor may occur as a result of neuromuscular irritability.

Post-surgical hypoparathyroidism may be transient, resolving within three to six weeks. Because it may be transient, calcium and vitamin D supplements are usually tapered slowly three to six weeks after surgery. Patients with a recurrence of hypocalcemia during the taper are more likely to have permanent hypoparathyroidism and require sustained oral supplementation. These patients also require monitoring of urinary calcium, serum calcium and serum phosphate weekly initially until a stable serum calcium concentration (at the low end of the normal range) is reached. This is followed by monitoring the levels at three- to six-month intervals. Subcutaneous administration of PTH 1-34 (teriparatide) and rhPTH 1-84 is effective in reducing the doses of oral calcium and vitamin D supplementation in patients with hypoparathyroidism. 

Our patient had a history of extensive neck surgery and radiation therapy due to laryngeal cancer in the distant past. During index hospitalization, he was found to have severe hypocalcemia with no classic signs or symptoms of hypocalcemia. He was initially given standard treatment (inhaled bronchodilator and steroid) but without significant improvement. After failing treatment for common causes, Endocrinology was consulted who recommended calcium gluconate infusion of 1 mg /kg/hr at 100 mg /hr, calcium carbonate 2000 mg PO Q8HR, low phosphate diet, to keep Ca > 7, ionized Ca > 5.5 and teriparatide SQ on discharge. Subsequently, his symptoms improved dramatically with liberation from the mechanical ventilator. Our patient also had vitamin D deficiency <12.5, which was replaced with a weekly 50,000 unit dose of vitamin D. IV calcium gluconate is usually preferred over calcium chloride due to the risk of skin necrosis if there is an IV infiltration during administration. A study done by Roy et al. has reported hypomagnesemia-induced hypocalcemia mimicking acute COPD exacerbation [8]. Also, Jain et al. have reported hypocalcemia-induced asthma exacerbation [9]. Our patient did not have any history of either asthma or COPD. He has never smoked in his life. 

## Conclusions

Early recognition of hypocalcemia induced bronchospasm is important in clinical practice to optimize management. Not many cases have been reported on hypocalcemia induced bronchospasm. This case provides an insight into a rare presentation of a common electrolyte disorder in a patient with no previous history of asthma or COPD.
